# Bidirectional crosstalk between the bone extracellular matrix and lysosomes in bone remodeling and osteoporosis

**DOI:** 10.3389/fendo.2025.1698404

**Published:** 2026-01-13

**Authors:** Chang Zhou, Xinyue Hu, Yichen Jing, Jiaheng Zhang, Jing Tao, Xinyi Ouyang, Jiaqian Tang, Guomin Zhang, Huiping Liu

**Affiliations:** College of Integrated Traditional Chinese and Western Medicine, Hunan University of Traditional Chinese Medicine, Changsha, Hunan, China

**Keywords:** bone ECM, lysosome, osteoblast, osteoclasts, osteocytes, osteoporosis

## Abstract

Osteoporosis is a systemic skeletal disorder characterized by progressive loss of bone mass and deterioration of microarchitectural integrity. Traditionally, its pathogenesis has been attributed primarily to an imbalance in the number and activity of osteoblasts and osteoclasts. However, emerging evidence has uncovered a critical bidirectional interdependence between the integrity of the extracellular matrix (ECM) and the functional homeostasis of the intracellular lysosomal system—an axis increasingly recognized as the “bone matrix–lysosome crosstalk.” Despite its apparent importance, the central role of this regulatory circuitry in bone homeostasis and the mechanisms through which it becomes disrupted under pathological conditions remain insufficiently defined.This review synthesizes current advances regarding the cell type–specific functions of lysosomes across distinct bone cell populations and further examines how the ECM, as a dynamic microenvironment, exerts reciprocal control over lysosomal biogenesis and activity. We highlight how the biochemical composition and biophysical properties of the ECM govern lysosomal acidification, metabolic coupling, and degradative capacity with remarkable precision. During the progression of osteoporosis, structural compromise of the ECM and lysosomal dysfunction reinforce one another, establishing a self-amplifying pathological loop that accelerates the collapse of the bone microenvironment. Recognizing this reciprocal deterioration, we propose that restoring the dynamic equilibrium of the “ECM–lysosome axis” may represent a mechanistic pivot for reversing osteoporotic degeneration. Interventions targeting lysosomal function, reconstructing the bone ECM, and employing nanomedicine-enabled organelle-specific delivery hold particular promise for advancing precision therapeutics in osteoporosis.

## 1 Introduction

Osteoporosis (OP) is a systemic metabolic bone disorder characterized by reduced bone mass, degenerative alterations in bone microarchitecture, and increased skeletal fragility, fundamentally driven by a long-standing imbalance in bone remodeling that favors bone resorption (1, 2). With the rapid acceleration of global population aging, the societal burden of OP continues to escalate (3). Epidemiological data indicate that more than 200 million people worldwide are currently affected, with prevalence increasing particularly among postmenopausal women and elderly men. The resulting fragility fractures markedly elevate mortality and disability rates and impose substantial medical and economic burdens (2, 3). Although existing anti-resorptive and anabolic therapies can partially slow disease progression, they remain insufficient to fully restore the homeostasis of the bone microenvironment, suggesting that the core pathogenic mechanisms underlying OP are not yet completely understood (4, 5).

Bone tissue homeostasis relies on the integrity and dynamic remodeling of the extracellular matrix (ECM, 6). The bone ECM consists of highly organized collagen fibrils, non-collagenous proteins, proteoglycans, and mineralized hydroxyapatite crystals, which together provide mechanical support and a signaling and adhesion microenvironment that governs the survival, migration, and functional states of osteoblasts, osteoclasts, and osteocytes (6). Emerging research increasingly highlights that the ECM is not a static scaffold but an active signaling integrator within the bone microenvironment. Through alterations in mechanical cues, biochemical composition, and nanoscale topology, the ECM triggers metabolic reprogramming and fate decisions in bone cells (7). Consequently, any disturbances to the ECM—such as abnormal collagen crosslinking, heterogeneous mineralization, reduced elastic modulus, or nanoscale structural damage—may serve as early pathological drivers of OP.

Importantly, the maintenance of ECM integrity is not solely determined by its inherent structure but requires continuous renewal orchestrated by intracellular degradation and biosynthetic systems within bone cells (8–10). Central to this process is the lysosome (11). Traditionally viewed as the terminal degradative compartment responsible for breaking down proteins, lipids, and damaged organelles, the lysosome is now recognized as a pivotal regulatory hub integrating nutrient sensing, energy metabolism, autophagic flux, extracellular secretion, and inter-organelle communication (12–14). In bone metabolism, osteoblasts rely on lysosome-mediated trafficking of matrix vesicles and recycling of ECM components to sustain mineralization (15); osteoclasts depend on lysosome-driven acidification and protease release to execute bone resorption (16); and osteocytes utilize the lysosome–autophagy axis to maintain lacunar–canalicular patency and preserve responsiveness to microenvironmental signals (17).

With advances in understanding lysosomal function, accumulating evidence suggests that OP is not merely the consequence of imbalanced osteoblastic and osteoclastic activities but also reflects a bidirectional dysregulation between ECM integrity and lysosomal homeostasis (15–17). Weakening of the ECM alters mechanical loading and transmembrane adhesion cues, thereby impairing lysosomal biogenesis, acidification, and metabolic coupling with other organelles (18). Conversely, lysosomal dysfunction disrupts ECM renewal, causes imbalanced matrix degradation, and compromises bone cell survival, establishing a pathological positive feedback loop that progressively damages bone microarchitecture (19).

Given the profound interdependence between the ECM and the lysosome in sustaining bone homeostasis, establishing a conceptual framework of “ECM–lysosome crosstalk” provides a new lens through which to understand the pathogenesis of OP. This review aims to systematically elucidate how the ECM and lysosome reciprocally regulate each other’s structure and function within bone tissue, identify the critical points at which this axis becomes disrupted during OP progression, and explore therapeutic strategies targeting this regulatory network, with the goal of guiding future precision interventions for OP.

## 2 Lysosomal regulation of bone ECM homeostasis

### 2.1 Lysosomal function core

The lysosome, long regarded as the cell’s central degradative system, performs far more complex and proactive roles than a conventional “waste-processing compartment.” It functions as a multidimensional regulatory platform essential for maintaining the homeostasis of the bone extracellular matrix (ECM) (16, 20). Its activities rely on tightly coordinated molecular machinery and highly orchestrated cell biological processes (21).

A fundamental function of the lysosome is its ability to degrade ECM components through its acidic lumen and extensive repertoire of hydrolytic enzymes. Hydroxyapatite (HA), the principal inorganic constituent of the bone matrix, undergoes chemical dissolution within the extracellular acidic microenvironment generated by polarized osteoclasts (22). Upon polarization, lysosome-derived V-ATPase and the ClC-7 chloride channel are redistributed to the ruffled border of osteoclasts (22), where massive proton extrusion reduces the pH of the resorptive lacuna to approximately 4.5 (23). This highly acidic environment destabilizes the HA crystal lattice and dissociates it into Ca²^+^ and PO_4_³^-^, completing inorganic mineral dissolution (24). Thus, HA degradation is essentially a lysosome-equipped, proton-pump–driven extracellular acidolysis reaction rather than an intralysosomal enzymatic process.

Importantly, only after acid dissolution of HA are type I collagen fibrils exposed to the resorption lacuna (25). Type I collagen accounts for nearly 90% of the organic ECM (26). Once exposed extracellularly, lysosome-derived cathepsin K (CatK), a cysteine protease, forms a specific complex with chondroitin sulfate, which is crucial for its ability to cleave triple-helical collagen (27). CatK further degrades the triple helix and telopeptide regions, effectively dismantling collagen fibrils (27). Genetic and clinical studies demonstrate that mutations in TCIRG1 (encoding the a3 subunit of V-ATPase) or CLCN7 (encoding ClC-7) prevent lacunar acidification to levels required for HA dissolution (28, 29). Consequently, minerals remain intact, and collagen cannot be accessed or degraded by CatK or other lysosome-derived proteases (30). Clinically, this results in abnormally elevated bone density with brittle, poorly functional cortical bone (“dense but fragile”), narrowed marrow cavities, impaired hematopoiesis, and cranial nerve compression (31, 32). At the cellular level, osteoclasts are present in normal numbers but exhibit defective polarization and lysosomal trafficking, rather than decreased cell abundance (16, 33). Altogether, these findings highlight the indispensable role of the lysosome in degrading bone ECM.

The lysosome also serves as a central platform for nutrient sensing and metabolic reprogramming. Amino acids and small peptides produced during ECM degradation are hydrolyzed in the lysosomal lumen and transported to the cytosol, providing essential nitrogen and carbon sources for the resynthesis of collagen and non-collagenous matrix proteins (34). Accumulating evidence shows that mTORC1 is the key molecular switch linking lysosomal nutrient signals to ECM biosynthesis programs (35). Under amino acid sufficiency, Rag GTPases and the Ragulator complex recruit mTORC1 to the lysosomal surface, positioning it near Rheb for full activation (36). In contrast, during amino acid deprivation, GAP factors such as GATOR1 promote GDP-loading of RagA/B, weakening Rag–Ragulator anchoring and rapidly detaching mTORC1 from the lysosomal membrane, leading to its inactivation (37, 38). This reversible localization, controlled by Rag nucleotide states, has been validated through live-cell imaging, immunofluorescence colocalization, and functional perturbation in multiple mammalian cell types, including HEK293T and MEFs (39).

Within this lysosomal nutrient-sensing module, SLC38A9 acts as both a transporter and sensor of amino acids (35, 40). It transports leucine and other amino acids to the cytosol while simultaneously modulating Ragulator–Rag conformation, ensuring proper mTORC1 recruitment and activation (40). Conversely, knockdown or inhibition of SLC38A9 blunts mTORC1 responsiveness to amino acids and impairs its lysosome-dependent activation (35), suggesting that amino acids recycled from ECM degradation are essential for sustaining mTORC1-driven anabolic metabolism. Functionally, mTORC1 directly regulates ECM synthesis in bone cells (41). Inhibition of mTORC1 (e.g., via rapamycin) reduces type I collagen (COL1A1) expression and hydroxyproline deposition *in vivo* and *in vitro* (42), and similar findings have been reported in BMSCs where COL1A1 levels change in response to rapamycin-modified autophagy (43). These studies position mTORC1 as a crucial anabolic node that senses lysosome-derived nutrients and drives ECM regeneration.

The lysosome further regulates ECM quality control and turnover through multiple forms of autophagy. Macroautophagy removes damaged organelles and protein aggregates via fusion of autophagosomes with lysosomes (44). Under stress, BMSCs show increased autophagosome abundance and formation of autolysosomes as observed by electron microscopy (45). When lysosomal acidification and degradation are inhibited by chloroquine, the autophagy substrate p62 accumulates and ECM secretion becomes dysregulated (46, 47). In parallel, chaperone-mediated autophagy (CMA) selectively degrades intracellular proteins carrying KFERQ-like motifs, relying on the lysosomal membrane receptor LAMP2A for substrate translocation (48). Studies show that CMA activity declines with age, whereas restoration of LAMP2A partially rescues aged cell function (49, 50). In bone, Lamp2a-deficient mice exhibit reduced vertebral trabecular bone mass, impaired osteoblast mineralization, and altered osteoclast-supporting signals (51). CMA defects in other tissues cause accumulation of oxidized proteins and disrupt proteostasis (52), suggesting that CMA deficiency may indirectly promote ECM abnormalities and bone fragility.

Lysosomal biogenesis and global lysosomal function are governed by transcription factor EB (TFEB). Decreased nuclear TFEB levels have been reported in osteoporotic bone tissues (53). In osteoclasts, TFEB activity is regulated by the TSC2–mTORC1–TFEB axis, maintaining lysosomal capacity and cellular homeostasis under hypoxia (54). TFEB dysfunction impairs lysosomal degradation, blocks clearance of mineralized matrix, and disrupts ECM remodeling and cartilage-to-bone transition (54). Conversely, TFEB knockdown inhibits autophagic flux (abnormal LC3-II/LC3-I ratios and p62 accumulation) and reduces osteogenic markers such as ALP and OCN (19, 55–57). Together, these findings establish TFEB-driven lysosomal function as a central upstream regulator of bone formation and ECM homeostasis.

**Figure 1:**
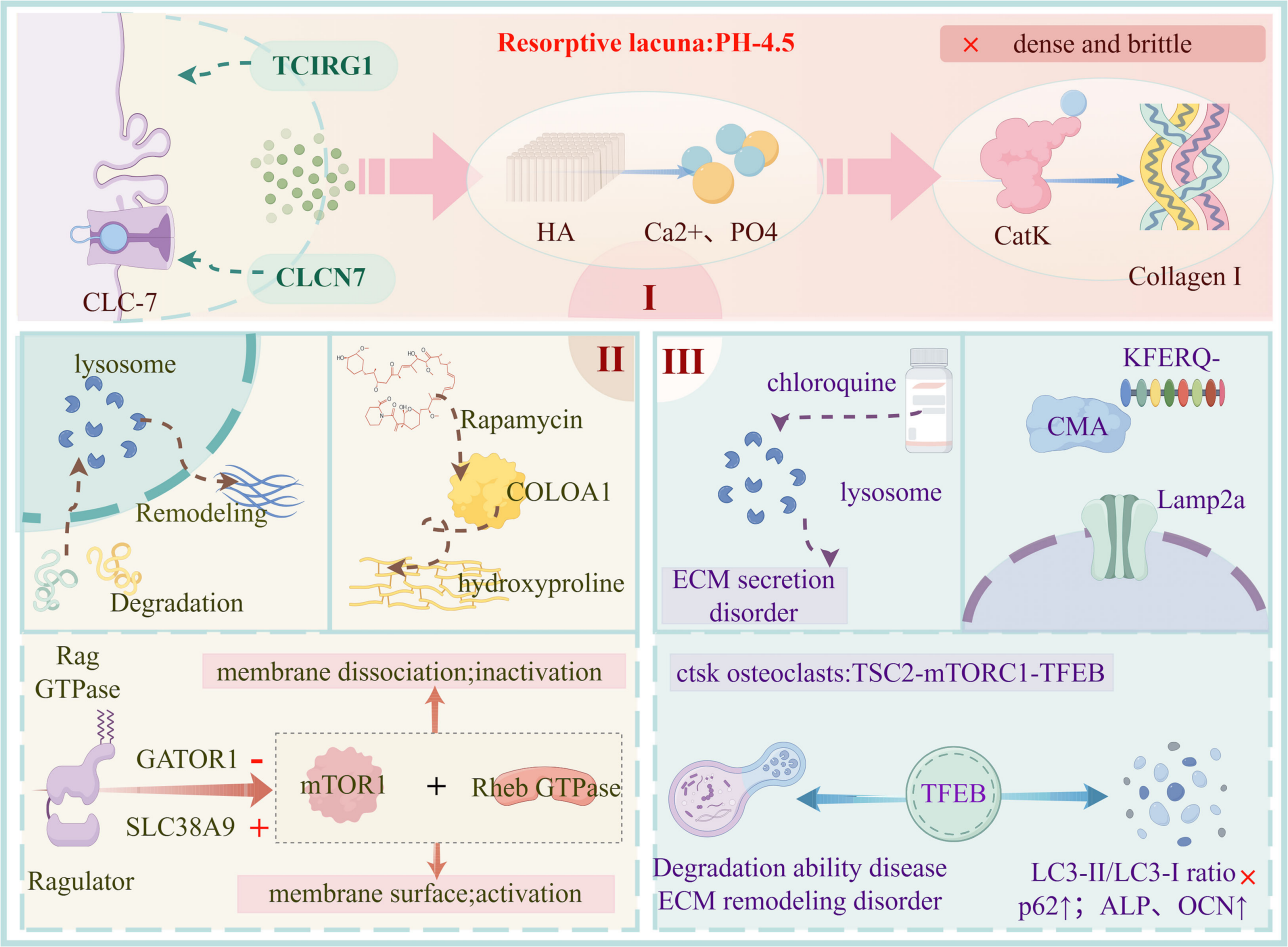
Central roles of lysosomal function. The figure illustrates the central roles performed by lysosomes in bone tissue, encompassing three major domains. (I) Extracellular matrix (ECM) degradation: Osteoclasts traffic lysosomal membranes enriched in vacuolar-type H^+^-ATPase (V-ATPase) and chloride channel 7 (ClC-7) to the ruffled border. This machinery acidifies the resorption lacuna to dissolve hydroxyapatite (HA), while exposed type I collagen is subsequently degraded by Cathepsin K(Cat K). Deficiencies in T-cell immune regulator 1 (TCIRG1), encoding a subunit of V-ATPase, or chloride channel 7 gene (CLCN7) impair acidification and thereby abolish ECM degradation. (II) Nutrient sensing and ECM regeneration: Degradation products are further processed within lysosomes and contribute to tissue remodeling. Rapamycin-mediated inhibition of mechanistic target of rapamycin complex 1 (mTORC1) reduces collagen deposition. Solute carrier family 39 member 9 (SLC39A9) and GTPase-activating protein toward Rags 1 (GATOR1) regulate the recruitment of mTORC1 to the lysosomal membrane via the Ragulator–Ras-related GTP-binding protein (Rag) complex, enabling or preventing its activation by the Ras homolog enriched in brain (Rheb) GTPase, thereby influencing ECM synthesis. (III) Autophagy-mediated processes: Chloroquine-induced inhibition of lysosomal function leads to disordered ECM secretion. Lysosome-associated membrane protein 2A (LAMP2A)–dependent chaperone-mediated autophagy (CMA) eliminates damaged intracellular components, while the transcription factor EB (TFEB) axis orchestrates lysosomal biogenesis and autophagy. ECM, extracellular matrix; V-ATPase, vacuolar-type H^+^-ATPase; ClC-7, chloride channel 7; CLCN7, chloride channel 7 gene; HA, hydroxyapatite; CatK, Cathepsin K; TCIRG1, T-cell immune regulator 1; mTORC1, mechanistic target of rapamycin complex 1; SLC39A9, solute carrier family 39 member 9; GATOR1, GTPase-activating protein toward Rags 1; Rag, Ras-related GTP-binding protein; Ragulator, LAMTOR1–5 complex; Rheb, Ras homolog enriched in brain; CMA, chaperone-mediated autophagy; LAMP2A, lysosome-associated membrane protein 2A; TFEB, transcription factor EB.

From substrate degradation to nutrient sensing and autophagy-mediated quality control, the lysosome operates through a highly coordinated, multifunctional network that exerts an active and indispensable role in maintaining the integrity and dynamics of the bone ECM (**Figure 1**).

### 2.2 Cell type-specific lysosomal regulation

Within the highly dynamic remodeling system of bone tissue, the dependence on lysosomal function varies substantially across different bone cell types. Although the lysosome is traditionally viewed as the intracellular degradative center, in the bone microenvironment it participates in far more diverse and specialized processes, including cell differentiation, ECM remodeling, initiation of mineralization, and intercellular communication. During the cyclical process of bone remodeling, osteoclasts, osteoblasts, and osteocytes respectively execute bone resorption, matrix production, and mechanosensation, and their lysosomal systems have therefore been “specialized” to accommodate distinct physiological demands. Such functional heterogeneity allows the lysosome to act not only as a degradative organelle but also as a critical signaling node in the bone remodeling network. Dissecting these cell-type–specific lysosomal regulatory modes is essential for understanding the fine-tuned mechanisms governing bone remodeling and the pathogenesis of osteoporosis.

In osteoclasts, the lysosomal system orchestrates a highly coordinated set of processes that enable efficient degradation of the bone ECM and constitute the core machinery of bone resorption. Upon attachment to mineralized bone surfaces, osteoclasts undergo pronounced cytoplasmic polarization, forming a ruffled border and a resorption lacuna that together create a sealed, highly acidified microenvironment known as the sealing zone (16). This microenvironment operates as an extracellular functional lysosome, formed through fusion of lysosomal vesicles with the bone-apposed plasma membrane (16). The acidic milieu is maintained by V-ATPase–driven proton pumping, which lowers the pH to levels suitable for mineral dissolution (23). Cathepsin K (CatK), one of the most critical lysosome-derived acidic proteases, specifically degrades collagen fibrils; its synthesis, activation, and targeted trafficking are precisely regulated to ensure effective ECM degradation (58). In Ctsk^-^/^-^ mice, overall bone remodeling persists, yet osteoclast lysosomal compartments accumulate undigested collagen fragments and exhibit severely compromised collagenolytic capacity, confirming the indispensable role of CatK in physiological bone resorption (59). Lysosomes not only participate in extracellular matrix degradation but also exert essential regulatory functions during osteoclast formation. The fusion and incorporation of lysosome-derived carrier vesicles furnish the bone-resorbing membrane with the acidification machinery and hydrolytic enzymes required to dissolve mineralized bone and degrade its organic matrix (60). Recent studies further demonstrate that disruption of endocytic–lysosomal trafficking or blockade of the forward transport of secretory lysosomes markedly impairs precursor-cell fusion and compromises osteoclast formation, underscoring the pivotal role of lysosome-dependent vesicular dynamics in driving osteoclastogenesis (61). Additionally, the RANKL pathway—a master regulator of osteoclast differentiation—directly modulates lysosomal enzyme expression, V-ATPase activity, and its subunit distribution via transcription factors such as NFATc1. These regulations exhibit clear stage- and dose-dependent patterns, indicating that lysosomal activity is dynamically tuned in response to remodeling demands rather than constitutively active (62). Disruption of lysosomal function leads to aberrant bone resorption—either excessive or insufficient—thereby destabilizing the balance between bone formation and resorption and predisposing to osteoporosis or osteosclerosis. Defining the spatial distribution, secretion dynamics, and regulatory networks of the osteoclast lysosome is therefore central to elucidating bone remodeling mechanisms and developing innovative anti-osteoporotic strategies.

Compared with the well-characterized role of the lysosome in osteoclast-mediated ECM degradation, recent studies have highlighted the importance of lysosomal regulation in osteoblasts and osteocytes for matrix synthesis, mineralization, and tissue homeostasis. During matrix mineralization, the lysosome performs functions that extend beyond classical degradation. Using nanoscale imaging, Iwayama et al. (63) demonstrated that mineralizing osteoblasts generate mineralizing vesicles (MVs), which accumulate within multivesicular bodies (MVBs) and undergo lysosome-dependent processing before extracellular release. These MVs contain amorphous calcium phosphate (ACP), deposit between collagen fibrils, and act as nucleation sites for mineral initiation. Pharmacological inhibition of lysosomal acidification or exocytosis markedly reduces MV secretion and mineral nodule formation, underscoring the indispensable role of the lysosome in initiating mineralization (63). In ECM synthesis, lysosomes regulate vesicle maturation, trafficking, and exocytosis, which are essential for the secretion of type I collagen and other ECM constituents (64). The autophagy–lysosome system in osteoblasts further maintains organelle quality control and supports differentiation. Dysregulated autophagy–lysosome activity impairs ECM production and disrupts mineralization rhythms, ultimately compromising bone-forming capacity (65, 66). Selective autophagy—particularly mitophagy—is crucial for long-term osteoblast function. Deficiency in Parkin-mediated mitophagy promotes accumulation of reactive oxygen species (ROS), accelerates osteoblast senescence, and markedly reduces matrix deposition (66), linking lysosomal quality-control pathways to age-related bone loss.

Osteocytes, which constitute 90–95% of all bone cells, reside deep within the lacuno–canalicular system (LCS), an extensive network of lacunae and canaliculi that allows intercellular connectivity and mechanotransduction (66). This unique architecture enables osteocytes to function as central orchestrators of mechanical sensing, ECM turnover, and mineral homeostasis (67). Accumulating evidence shows that the ECM-regulating capacity of osteocytes relies heavily on the lysosome–autophagy axis (68). As “microenvironmental custodians,” osteocytes perform perilacunar/canalicular remodeling (PLR) to maintain LCS morphology and patency (69). This process requires lysosomal storage and secretion of hydrolytic enzymes, including CatK (69). Conditional knockout studies demonstrate that suppression of osteocyte-derived CatK blocks lactation-associated PLR and prevents bone loss (70), indicating that osteocytes actively remodel ECM rather than relying solely on osteoclasts. Autophagy–lysosome pathways are equally essential for osteocyte quality control and ECM homeostasis. Basal autophagy clears damaged mitochondria and protein aggregates, sustaining osteocyte metabolic fitness and mechanosensitivity (17). Impaired autophagic flux or abnormal lysosomal acidification causes rapid cellular injury, apoptosis, and functional decline. Mechanical loading induces autophagy-dependent RANKL secretion from osteocytes, promoting osteoclastogenesis and facilitating matrix removal (71), linking osteocyte health directly to bone structural integrity. Furthermore, the lysosome–endosome system regulates the biogenesis and secretion of extracellular vesicles (EVs) and matrix vesicles, whose cargoes—proteases, miRNAs, and signaling peptides—dynamically modulate ECM synthesis and resorption in neighboring osteoblasts, osteoclasts, and BMSCs (72). These findings position osteocyte lysosomal function as a pivotal hub that integrates inward degradative processes with outward signaling cues, thereby maintaining bone quality and resilience. Dysfunction of the lysosome or autophagy in osteocytes simultaneously disrupts intracellular homeostasis and ECM-directed communication, leading to LCS deterioration, excessive pro-resorptive signaling, and ultimately compromised bone strength. Thus, the osteocyte lysosome–autophagy axis represents a promising but underexplored therapeutic target beyond traditional osteoclast- and osteoblast-centered strategies.

In summary, from osteoclast-dependent lysosome-driven bone resorption, to osteoblasts utilizing lysosomes for matrix transport and mineralization initiation, to osteocytes employing the lysosome-autophagy axis to maintain lacuno-canalicular system integrity and regulate intercellular signaling, the role of lysosomes in distinct bone cells extends far beyond that of a traditional intracellular degradation organ. Instead, it exhibits a highly cell-type-specific functional landscape. These distinctions not only govern the rhythm and efficiency of bone remodeling but also constitute the key molecular basis for diseases such as osteoporosis. Future in-depth analysis of lysosomal distribution patterns, dynamic regulation, and their upstream signaling networks within distinct bone cells will offer novel perspectives on cross-cellular regulation of bone remodeling. This research will also provide crucial theoretical foundations for developing precision therapeutic strategies targeting lysosomal function (**Table 1**).

**Table 1:** Cell–type–specific lysosomal regulation in bone remodeling.

Bone cell type	Lysosome-dependent biological function	Key mechanism (one per row)	Reference
Osteoclasts	ECM degradation	Fusion of lysosomal vesicles with bone-apposed plasma membrane forms a specialized extracellular “functional lysosome”.	(16)
Osteoclasts	ECM degradation	V-ATPase pumps protons to generate an acidic pH required for mineral dissolution.	(23)
Osteoclasts	Collagen degradation	Cathepsin K (CatK) digests collagen fibrils during resorption.	(58)
Osteoclasts	Collagen degradation	Ctsk^-^/^-^ mice accumulate undigested collagen in lysosomes, confirming CatK necessity.	(59)
Osteoclasts	Regulation of differentiation	RANKL–NFATc1 axis modulates lysosomal enzyme expression and V-ATPase distribution.	(62)
Osteoblasts	Matrix mineralization	Mineralizing vesicles (MVs) are processed in MVBs via a lysosome-dependent pathway before secretion.	(63)
Osteoblasts	ECM protein secretion	Lysosomes regulate vesicle maturation and exocytosis for type I collagen secretion.	(64)
Osteoblasts	Maintenance of ECM production	Autophagy–lysosome dysfunction reduces ECM synthesis and disrupts mineralization rhythm.	(65, 66)
Osteoblasts	Redox homeostasis	Parkin-dependent mitophagy prevents ROS accumulation and suppresses osteoblast senescence.	(67)
Osteocytes	PLR (perilacunar/canalicular remodeling)	Lysosomes store and secrete hydrolytic enzymes such as CatK for ECM remodeling.	(69)
Osteocytes	PLR	CatK deletion in osteocytes blocks lactation-associated PLR and prevents bone loss.	(70)
Osteocytes	Mechanosensation	Basal autophagy maintains mitochondrial quality control and osteocyte mechanosensitivity.	(17)
Osteocytes	Regulation of osteoclastogenesis	Mechanical loading induces autophagy-dependent RANKL release from osteocytes.	(71)
Osteocytes	Intercellular communication	Lysosome–endosome pathways regulate EV biogenesis and cargo loading.	(72)

ECM, Extracellular Matrix; PLR, Perilacunar/Canalicular Remodeling; MV, Mineralizing Vesicle; MVB, Multivesicular Body; BMSC, Bone Marrow–Derived Mesenchymal Stem Cell; CatK, Cathepsin K; CTSK, Cathepsin K Gene; V-ATPase, Vacuolar-type H^+^-ATPase; LC3, Microtubule-Associated Protein 1A/1B-Light Chain 3; ATG2, Autophagy-Related Protein 2; ATG7, Autophagy-Related Protein 7; RUFY4, RUN and FYVE Domain–Containing Protein 4; Rab7, Ras-Related Protein Rab-7; LAMP2, Lysosome-Associated Membrane Protein 2; SNX10, Sorting Nexin 10; TCIRG1, T Cell Immune Regulator 1 (H^+^-ATPase Subunit); CLCN7, Chloride Channel 7; TNFRSF11A, Tumor Necrosis Factor Receptor Superfamily Member 11A; IDUA, α-L-Iduronidase; VPS4A, Vacuolar Protein Sorting-Associated Protein 4A; TMEM175, Transmembrane Protein 175; RANKL, Receptor Activator of Nuclear Factor κB Ligand; NF-κB, Nuclear Factor kappa-B; NFATc1, Nuclear Factor of Activated T Cells 1; WNT, Wnt Signaling Pathway; PKA, Protein Kinase A; PTH, Parathyroid Hormone; PTHrP, Parathyroid Hormone– Related Peptide; 1,25(OH)_2_D, 1,25-Dihydroxyvitamin D; ROS, Reactive Oxygen Species; HA, Hydroxyapatite; DHAP, Dihydroxyacetone Phosphate; ATP, Adenosine Triphosphate; cAMP, Cyclic Adenosine Monophosphate; PO_4_³^-^, Phosphate Ion; GC, Glucocorticoid; MPSIH, Mucopolysaccharidosis Type I H; BV/TV, Bone Volume/Tissue Volume; BMD, Bone Mineral Density.

## 3 Bone ECM affects the realization of lysosomal function

### 3.1 Chemical microenvironment of the bone matrix: a multidimensional regulatory network governing lysosomal biogenesis

The chemical microenvironment of the bone matrix is far more than a static structural scaffold. Rather, it constitutes a dynamic, multidimensional signaling system composed of structural components (minerals, collagen), instructive biochemical signals (growth factors, cytokines), regulatory cues (enzymes, pH), and energy and metabolic inputs (73–76). By integrating these biochemical dimensions, the bone matrix provides precise guidance for lysosomal biogenesis and function, positioning itself as a central orchestrator of bone remodeling.

The structural components of the matrix—primarily inorganic minerals and organic substrates—define the initial physicochemical landscape in which lysosomes operate (77). Inorganic minerals are predominantly arranged as hydroxyapatite crystals, conferring mechanical strength to the skeleton (78). During bone resorption, osteoclasts generate a sealed zone and ruffled border (16), where V-ATPase localizes to the ruffled border membrane to pump protons outward, thereby dissolving hydroxyapatite and releasing calcium and phosphate (79, 80). Elevated extracellular Ca²^+^ can bind the lysosomal membrane channel TRPML1 and enhance membrane permeability, triggering Ca²^+^ efflux and activating the calcineurin–TFEB axis (81). Lysosomal biogenesis is centrally regulated by TFEB, whose nuclear activation state dictates the transcriptional program and functional maturation of the lysosomal network (82). Conversely, high phosphate concentrations—especially in the form of calcium–phosphate complexes (CPPs)—can be internalized and accumulate within late endosomes/lysosomes, neutralizing luminal acidity, inhibiting hydrolase activity, and impairing matrix-fragment degradation (83–85).

Collagen type I, which constitutes over 90% of the organic matrix, not only provides a scaffold for mineral deposition but also shapes lysosomal signaling through its degradation fragments. These fragments activate integrin α2β1 and stimulate downstream FAK/mTORC1 signaling, thereby relieving TFEB cytoplasmic retention and promoting its nuclear entry (36, 86, 87). Through these mechanisms, structural components of the matrix directly set the biochemical “background” for lysosomal activity.

Embedded within the matrix, bioactive molecules form an additional regulatory layer that translates matrix degradation signals into precise instructions for lysosomal remodeling. Growth factors such as BMPs and TGF-β are stored in a latent state bound to matrix proteins including osteopontin and fibronectin. They are activated during osteoclast-mediated acidification and matrix dissolution (74, 88). Activated TGF-β triggers intracellular Ca²^+^ elevation and stimulates the calmodulin–calcineurin axis, promoting dephosphorylation and nuclear translocation of TFEB/TFE3 and enhancing transcription of lysosomal and autophagy-related genes (89, 90). Likewise, BMP2 activates the BMPR–TAK1 pathway, which may cooperate with the TAB/RCAN complex to fine-tune calcineurin activity and further amplify TFEB-driven lysosomal biogenesis and acidification (91).

Cytokines secreted by bone matrix–embedded cells constitute yet another tier of regulation. RANKL binds the receptor RANK on osteoclast precursors, recruits TRAF6, and activates NF-κB and MAPK signaling, ultimately inducing the expression and nuclear localization of the master transcription factor NFATc1 (92, 93). NFATc1 binds promoters of lysosomal genes—including cathepsin K, LAMP2, and V-ATPase subunits—constructing the lysosomal apparatus required for efficient bone resorption (16, 62). In addition, RANKL activates PKCβ-dependent phosphorylation pathways that enhance TFEB activation, driving lysosomal maturation during osteoclast differentiation (94). Loss of TFEB or PKCβ suppresses lysosomal gene expression and increases bone mass, underscoring the essential role of the RANKL–PKCβ–TFEB axis in controlling lysosomal biogenesis and bone resorption (94). Conversely, osteoblast-derived OPG competitively binds RANKL and attenuates NFATc1-mediated lysosomal remodeling (93).

The enzymatic milieu and pH gradients created within the bone matrix function as rapid and direct regulatory tools that modulate lysosomal activity and membrane stability. Acidification at the osteoclast ruffled border reflects a dynamic equilibrium constrained by matrix chemistry rather than a unidirectional proton-pumping process (16, 79, 95). V-ATPase-driven proton extrusion dissolves hydroxyapatite, releasing Ca²^+^ and PO_4_³^-^, which in turn modify extracellular ionic strength and buffer capacity, thereby influencing lysosomal membrane electrochemical stability and fusion/exocytosis efficiency (16, 96). The stability of lysosomal membranes requires a sustained proton gradient and correct folding/glycosylation of membrane proteins such as LAMP1, LAMP2, and H^+^-dependent chaperones (97, 98). Disruption of luminal acidification leads to lysosomal membrane permeabilization (LMP), leakage of cathepsins B/D, and downstream stress responses (99–101). Thus, the pH microenvironment defined by the matrix not only determines catalytic efficiency but also regulates lysosomal membrane integrity and secretory rhythm. This matrix–lysosome interplay reveals new molecular foundations for pathological bone resorption and identifies potential therapeutic windows targeting pH-lysosome homeostasis.

Lysosomal biogenesis, membrane trafficking, and maintenance of luminal acidity are energy-demanding processes. The chemical microenvironment of the matrix provides critical metabolic support by reshaping cellular metabolic programs and energy signaling pathways (75, 102, 103). Bone matrix cells—particularly osteoblasts and osteoclasts—dynamically adjust metabolic preferences in response to local oxygen tension, ionic composition, and lactate levels (7, 104). Hypoxia in remodeling or repair sites stabilizes HIF-1α, shifting metabolism from oxidative phosphorylation toward glycolysis to sustain ATP production under low O_2_ (105). Glycolytic ATP supports energy-intensive systems such as V-ATPase and lysosomal transport machinery and also regulates TFEB nuclear translocation via the AMPK–mTORC1 axis, thereby promoting lysosomal biogenesis and autophagic flux (106–108). Glycolytic by-products, including lactate and associated protons, help maintain local pH conditions favorable for lysosomal enzyme activity (109). Moreover, the skeleton acts as an endocrine organ; osteocalcin (OCN) enhances systemic metabolic efficiency by stimulating insulin signaling in β-cells and skeletal muscle, indirectly supporting the metabolic demands of osteoblasts (110). Through this integration of local and systemic metabolic cues, the matrix microenvironment serves not only as a reaction field for lysosomal activity but also as a metabolic power source.

**Figure 2:**
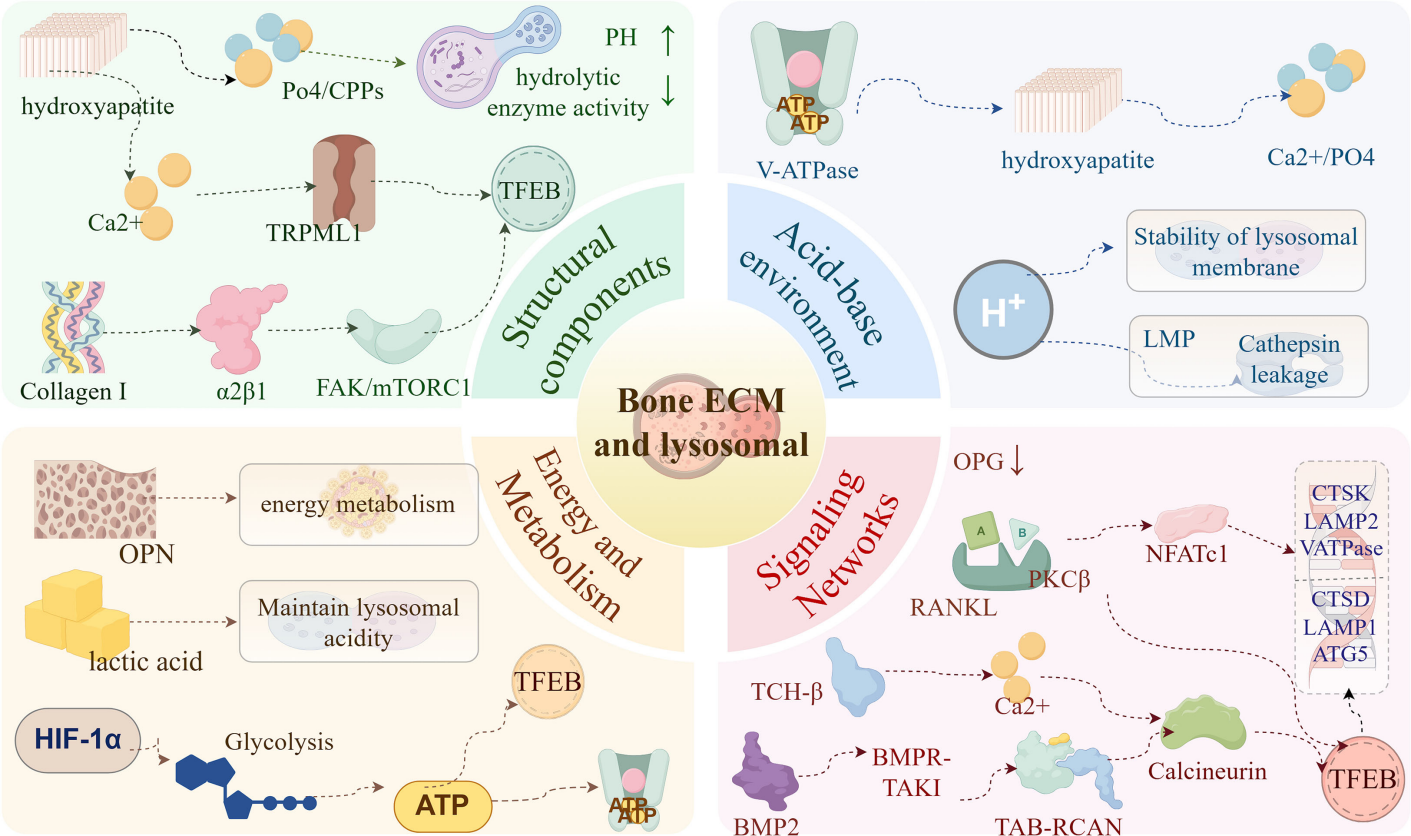
Bone matrix microenvironment: a multidimensional regulatory network governing lysosomal biogenesis the schematic illustrates how the structural components of the bone matrix, the acidic microenvironment, signaling networks, and energy–metabolic cues collectively regulate lysosomal activity and transcription factor EB (TFEB)–dependent lysosomal biogenesis (1). Structural components:Hydroxyapatite dissolution releases calcium ions (Ca²^+^) and phosphate ions (PO_4_³^-^), which activate Ca²^+^ signaling through transient receptor potential mucolipin 1 (TRPML1) and promote the nuclear translocation of TFEB. Phosphate ions and calcium phosphate particles (CPPs) elevate lysosomal pH and suppress lysosomal hydrolase activity. Type I collagen regulates TFEB through the α2β1 integrin–focal adhesion kinase (FAK)–mechanistic target of rapamycin complex 1 (mTORC1) axis. (2) Acidification and pH regulation:Vacuolar-type H^+^-ATPase (V-ATPase) maintains lysosomal acidity, facilitates hydroxyapatite dissolution, and establishes a Ca²^+^/PO_4_³^-^ feedback loop. Elevation of lysosomal pH leads to membrane destabilization and lysosomal membrane permeabilization (LMP), resulting in the leakage of Cathepsins into the cytosol. (3) Signaling networks:Transforming growth factor-β (TGF-β) and bone morphogenetic protein 2 (BMP2) activate TFEB via the Ca²^+^–calcineurin pathway. Receptor activator of nuclear factor-κB ligand (RANKL)–nuclear factor of activated T cells 1 (NFATc1) signaling upregulates lysosomal and osteoclastic genes, whereas osteoprotegerin (OPG) exerts inhibitory effects. (4) Energy and metabolic cues:Hypoxia–hypoxia-inducible factor 1α (HIF-1α) enhances glycolysis and adenosine triphosphate (ATP) supply to support V-ATPase activity. Lactate and protons (H^+^) help maintain lysosomal acidity. Osteocalcin (OCN) links systemic energy metabolism to lysosomal function in bone cells. ATP, adenosine triphosphate; BMP2, bone morphogenetic protein-2; Ca²^+^, calcium ion; CPPs, calcium phosphate particles; FAK, focal adhesion kinase; H^+^, proton; HIF-1α, hypoxia-inducible factor-1α; LMP, lysosomal membrane permeabilization; mTORC1, mechanistic target of rapamycin complex-1; NFATc1, nuclear factor of activated T cells 1; OCN, osteocalcin; OPG, osteoprotegerin; PO_4_³^-^, phosphate ion; RANKL, receptor activator of nuclear factor-κB ligand; TGF-β, transforming growth factor-β; TFEB, transcription factor EB; TRPML1, transient receptor potential mucolipin-1; V-ATPase, vacuolar-type H^+^-ATPase.

Collectively, the chemical microenvironment of the bone matrix forms a multilayered, dynamic regulatory network that integrates structural cues, biochemical signals, regulatory tools, and metabolic inputs to orchestrate lysosomal biogenesis and functional output. Future research should employ advanced platforms—such as real-time single-organelle imaging and biomimetic matrix systems—to delineate the dynamic interactions among these dimensions under physiological and pathological conditions. These insights are expected to uncover precise molecular targets and innovative therapeutic strategies aimed at modulating lysosomal function to treat osteoporosis and other skeletal disorders. (**Figure 2**).

### 3.2 Mechanistic regulation of lysosomal function by the physical properties of the extracellular matrix

The physical attributes of the extracellular matrix (ECM) constitute a critical regulatory dimension for lysosomal function, complementing the chemical signaling framework described above. Emerging evidence indicates that ECM stiffness, topographical organization, and piezoelectric properties profoundly influence lysosomal biogenesis, spatial distribution, trafficking dynamics, and degradative capacity through mechanotransduction, cytoskeletal remodeling, and nanoscale force responsiveness. These insights establish a new conceptual foundation for understanding skeletal homeostasis and the pathological mechanisms underlying osteoporosis.

YAP/TAZ act as core mechanosensitive transcriptional regulators within the bone microenvironment, with their phosphorylation status and nuclear localization directly responding to ECM stiffness. At the cellular level, stiff substrates are sensed via integrin–focal adhesion (FA) complexes, where integrin clustering and FA maturation activate the FAK/Src–RhoA/ROCK cascade, promoting actin stress fiber assembly and establishing a robust intracellular tension network (10, 111, 112). This structural–mechanical coupling enables efficient transmission of rigidity cues to YAP/TAZ activation. Under stiff ECM conditions, YAP/TAZ undergo dephosphorylation and nuclear translocation (103, 113), where they interact with TFE3 or TFEB to form transcriptional complexes that initiate lysosomal and autophagy-related gene expression (114). Nuclear YAP/TAZ activation further modulates mTORC1 assembly and activity at the lysosomal membrane, thereby regulating lysosomal acidification and proteolytic efficiency (115, 116).

Mechanotransduction also induces metabolic reprogramming. Upon YAP/TAZ activation, mesenchymal stem cells exhibit enhanced glycolysis and mitochondrial oxidative phosphorylation, resulting in elevated ATP production that fuels V-ATPase-dependent proton pumping in lysosomes (103, 106). Concurrently, mechanical stimulation leads to the accumulation of metabolites such as succinate and lactate (117, 118), which function not only as metabolic intermediates but also as epigenetic regulators of lysosome-related transcription factors (119, 120). For example, succinate inhibits the demethylase FTO, increasing m6A modification of osteogenic regulators such as Runx2 and promoting coordinated activation of osteogenic gene expression and lysosomal function (15, 121, 122). Collectively, ECM stiffness engages YAP/TAZ as central mechanosensors to orchestrate a hierarchical regulatory cascade—ranging from cytoskeletal remodeling to metabolic control—that links mechanical inputs to lysosomal function. This mechanotransduction–transcription–metabolism axis reveals a multilayered framework through which the bone microenvironment regulates intracellular degradative systems and maintains skeletal homeostasis.

The ECM also possesses hierarchical topographical features, with collagen fibrils exhibiting a periodic banding pattern (~67 nm) and intertwining with hydroxyapatite crystals in defined orientations to form a highly organized composite structure (123). These topographical attributes serve not only as structural scaffolds but also as regulatory platforms for organelle function (124), particularly during lysosome-dependent matrix remodeling. Cells detect and interpret ECM topography via integrin–FA complexes, transmitting these cues to the RhoA/ROCK axis to modulate cytoskeletal tension (125–127). Variations in collagen alignment and microdomain stiffness reshape FAK-mediated mechanotransduction, altering microtubule stability and actin filament dynamics (128, 129). This spatial reorganization of cytoskeletal tension influences lysosomal transport along microtubules, affecting their subcellular distribution and interactions with mitochondria, which are critical for energy and signaling homeostasis (130, 131).

Recent advances in multiscale organelle mechanobiology demonstrate that lysosomal motility, mitochondria–lysosome contact frequency, and membrane fusion probability are highly sensitive to external mechanical inputs. On stiff or highly aligned substrates, increased cytoskeletal tension and traction forces promote lysosomal clustering near FA-rich regions and the cell periphery, facilitating mechanically coupled functions such as membrane fusion and exocytosis (131, 132). In contrast, soft or topographically disordered ECM leads to more diffuse lysosomal distribution and a higher prevalence of mitochondria-associated lysosomal subpopulations (131), altering lysosomal activity (133, 134). This form of “spatial reprogramming” underscores the mechanosensitivity of lysosomes and highlights ECM topology as a determinant of local enzymatic release and degradative precision.

The piezoelectric properties of the ECM create a unique mechano-electrical coupling interface that links physical stimuli to cellular metabolic and signaling responses (135–137), offering a non-chemical mechanism for shaping lysosomal function. As a natural electromechanical converter, collagen generates piezoelectric dipoles upon mechanical deformation, which can drive hydroxyapatite nucleation even in the absence of osteoblasts (138). Conversely, hydroxyapatite enhances the mechanical responsiveness of collagen fibrils through structural reinforcement, promoting alignment of piezoelectric domains and amplifying overall piezoelectric output (139). Mechanical loading or physiological body movement can induce localized electrical potentials within bone tissue or engineered piezoelectric substrates, selectively activating mechanosensitive ion channels such as Piezo1 and triggering rapid intracellular Ca²^+^ transients on the millisecond–second scale (140, 141). These Ca²^+^ signals activate the calcineurin–TFEB pathway, thereby influencing lysosomal biogenesis (81).

**Figure 3:**
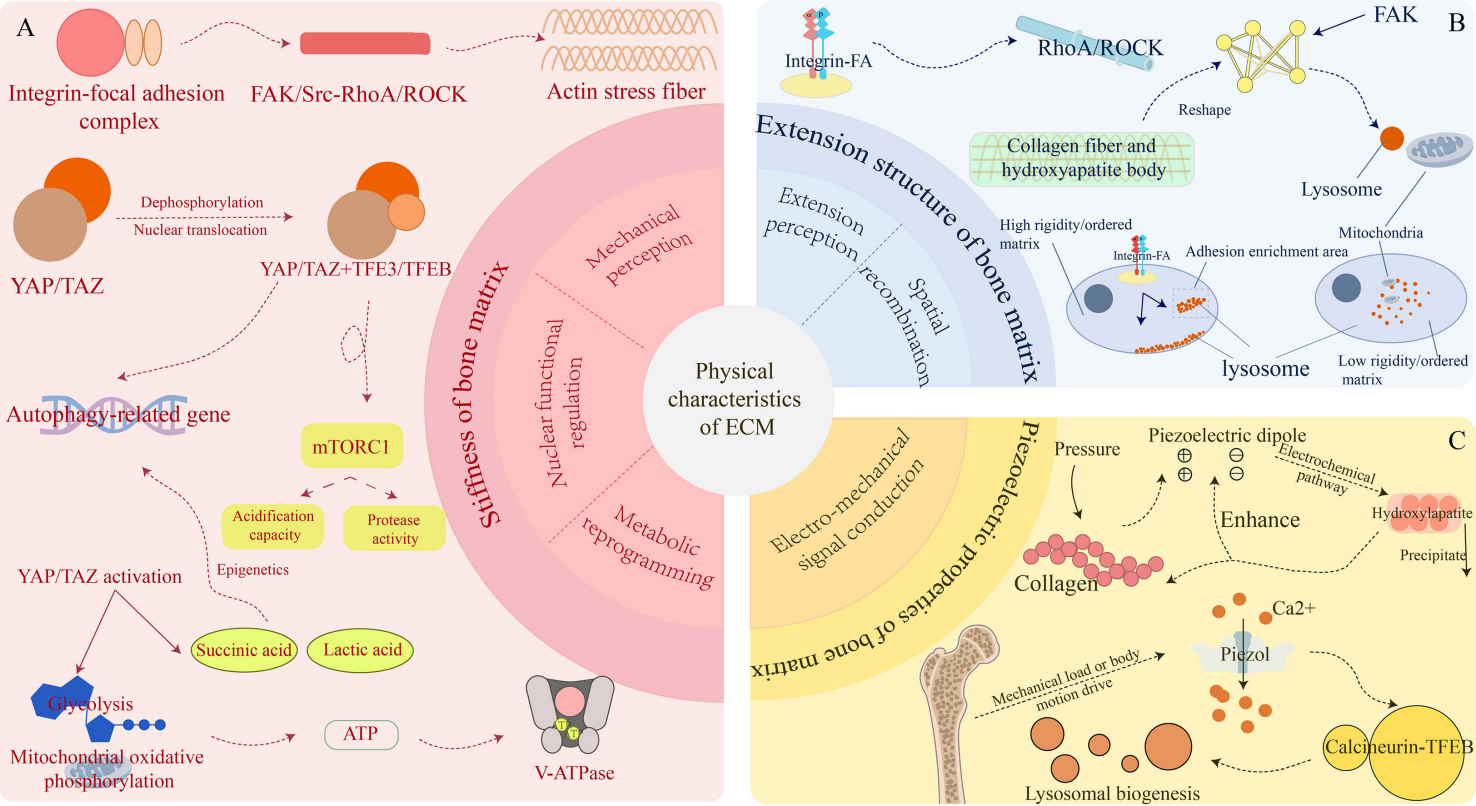
Regulatory mechanisms by which extracellular matrix (ECM) physical properties govern lysosomal function this figure illustrates how three key physical attributes of the bone extracellular matrix—stiffness, extension structure, and piezoelectric property—integrate to regulate lysosomal positioning, acidification, and transcription factor EB (TFEB)–dependent lysosomal biogenesis. **(A)** Matrix stiffness.Sensing of matrix stiffness activates integrin–focal adhesion (FA) complexes, which in turn stimulate focal adhesion kinase (FAK)/Src and RhoA/Rho-associated kinase (ROCK) signaling, promoting the formation of actin stress fibers. These events drive the dephosphorylation and coordinated nuclear translocation of Yes-associated protein (YAP)/transcriptional coactivator with PDZ-binding motif (TAZ) together with transcription factor E3 (TFE3)/TFEB. Downstream effects include modulation of mechanistic target of rapamycin complex 1 (mTORC1), enhancement of lysosomal acidification capacity, increased protease activity, metabolic reprogramming (including lactate and succinate production), and upregulation of autophagy-related gene expression. **(B)** Extension structure of the ECM. The spatial extension architecture of the ECM influences lysosomal distribution. High-stiffness or highly ordered collagen–hydroxyapatite composite structures strengthen integrin clustering and focal adhesion remodeling, facilitating lysosomal accumulation along cytoskeletal traction pathways. In contrast, low-stiffness or disordered ECM structures cause lysosomal dispersion and disrupt coordinated mitochondrial metabolism. **(C)** Piezoelectric properties of the bone matrix. The piezoelectric effect of bone arises from periodic mechanical loading on collagen fibrils, generating dipole signals. Piezo-type mechanosensitive ion channel component 1 (Piezo1) senses mechanically induced or movement-driven calcium ion (Ca²^+^) influx, activating the calcineurin–TFEB pathway and promoting lysosomal biogenesis. Hydroxyapatite deposition further amplifies the piezoelectric response, establishing a positive feedback loop linking ECM, Ca²^+^ signaling, and lysosomal function. ECM, extracellular matrix; FA, focal adhesion; FAK, focal adhesion kinase; Src, proto-oncogene tyrosine-protein kinase Src; RhoA, Ras homolog family member A; ROCK, Rho-associated kinase; YAP, Yes-associated protein; TAZ, transcriptional coactivator with PDZ-binding motif; TFE3, transcription factor E3; TFEB, transcription factor EB; mTORC1, mechanistic target of rapamycin complex 1; Piezo1, Piezo-type mechanosensitive ion channel component 1; Ca²^+^, calcium ion; HA, hydroxyapatite; lactate, lactic acid; succinate, TCA-cycle intermediate.

In summary, the physical properties of the ECM constitute a multidimensional regulatory network that governs lysosomal function. These insights not only deepen the current understanding of bone biology but also provide a theoretical basis for developing therapeutic strategies that target the physical microenvironment. Future research should focus on elucidating how diverse physical cues are integrated into unified cellular responses and on designing intelligent biomaterials capable of dynamically guiding bone regeneration, thereby opening new avenues for the prevention and treatment of osteoporosis and related skeletal disorders (**Figure 3**).

## 4 The interaction mechanism between lysosomes and bone ECM in osteoporosis

### 4.1 Mechanisms underlying bone resorption

Aberrantly enhanced bone resorption is a fundamental pathological basis of osteoporosis, driven primarily by dysregulation of how osteoclast lysosomal systems recognize bone ECM, direct polarized secretion, and re-internalize and process degradation products (16). Among these processes, cathepsin K (CatK; encoded by CTSK) is the pivotal collagenolytic enzyme within lysosomes and serves as a decisive determinant of bone resorption. Cellular studies demonstrate that maximal inhibition of CatK completely blocks the osteoclast transition from pit-type to trench-type resorption, markedly reducing trench-like erosion while increasing pit-like corroded areas. These findings indicate that CatK activity directly shapes osteoclast selectivity toward distinct ECM structural types (142). In estrogen-deficient mouse models, delayed trabecular mineralization, disorganized collagen fibrils, and heightened osteoclast activity coincide with pronounced CTSK upregulation, supporting the notion that hyperactivated lysosomal proteolysis is a key driver of ECM degradation in osteoporosis (143). Conversely, CTSK knockout results in increased trabecular number and thickness, denser microarchitecture, and a significant elevation in bone volume fraction (BV/TV), representing a typical phenotype of impaired bone resorption and secondary bone mass accumulation (144). However, despite greater stiffness and strength, Ctsk^-^/^-^ bone exhibits reduced deflection prior to fracture, displaying a “stiffer-but-more-brittle” mechanical profile. This suggests that the absence of CatK-mediated organic matrix remodeling compromises energy absorption and elasticity in bone tissue (144).

Clinical studies further reinforce the centrality of CatK in bone resorption control. The CatK inhibitor odanacatib significantly reduces biochemical markers of bone resorption and increases bone mineral density (145, 146), with its efficacy closely correlated with reduced CatK activity (147, 148). CatK is also persistently upregulated in osteoarthritic tissues (149). Other inhibitors, such as ONO-5334, exhibit sustained suppression of bone resorption (150, 151). Nevertheless, several CatK inhibitors failed to reach clinical application owing to cardiovascular and dermatologic adverse events observed in trials (148). Collectively, these findings establish CatK as the terminal effector molecule of osteoclast lysosomal ECM degradation, and its aberrant activation as a prominent hallmark of excessive bone resorption and osteoporosis progression.

Beyond enzymatic degradation, lysosomal membrane trafficking and localization mechanisms are equally essential in governing ECM breakdown. The membrane trafficking protein RUFY4, via its RUN and FYVE domains, mediates protein–membrane interactions and directs lysosomal transport along microtubules to the osteoclast ruffled border, thereby coordinating lysosomal positioning, fusion, and cargo release (152). Recent work identifies RUFY4 as a critical regulatory hub in bone resorption: its expression is induced by the RANKL–NF-κB p65–NFATc1 pathway, and RUFY4 deficiency leads to decoupling of the Rab7–LAMP2 complex, reduced acidic lysosome abundance, diminished CatK secretion, impaired collagen degradation, and increased bone density accompanied by defective remodeling (20). Thus, RUFY4 acts as a molecular axis coupling ECM-derived cues to lysosomal trafficking, indispensable for osteoclast resorptive function.

From the perspective of membrane-domain construction, SNX10-mediated vesicular trafficking also plays an essential role in bone resorption. Evidence indicates that SNX10 controls the formation and transport of distinct membrane compartments, maintaining the integrity of the osteoclast plasma membrane–ruffled border axis (153). Various autosomal recessive osteopetrosis (ARO) models resulting from SNX10, TCIRG1, or CLCN7 mutations consistently exhibit defective ruffled border formation and impaired acidification capacity in osteoclasts, demonstrating that despite differing molecular roles, these genes converge functionally to disrupt the ruffled-border-centered “acidification–secretion” system critical for ECM degradation (23, 79, 154, 155). Convergent findings from human genetics, animal models, and cellular studies related to the SNX10 (R51Q) mutation firmly establish SNX10 as a key node required for lysosomal membrane functions during osteoclast-mediated resorption.

Histological evidence from human osteopetrosis further highlights the distinct contributions of lysosomal function to normal ECM mineralization. In patients harboring TCIRG1 mutations, undecalcified iliac crest biopsies consistently reveal abundant osteoid accumulation and significantly reduced mineralization, indicating that defects in the lysosomal H^+^-ATPase subunit impair lysosomal acidification and directly induce osteomalacia (156). In contrast, patients with CLCN7 or TNFRSF11A mutations exhibit impaired bone resorption but preserved ECM mineralization, suggesting that lysosomal acidification and lysosomal ion homeostasis represent separable regulatory modules of ECM processing (156). This distinction demonstrates that adequate lysosomal acidification is indispensable for proper mineralization cycles, whereas ion flux regulation primarily affects the efficiency of bone resorption rather than mineralization itself.

Clinical evidence from lysosomal storage disorders also supports the central role of lysosome–matrix interactions in skeletal homeostasis. In mucopolysaccharidosis type I H (MPSIH), deficiency of IDUA leads to lysosomal and extralysosomal accumulation of glycosaminoglycans (GAGs) in osteoblasts and other cells, disturbing ECM metabolism and connective tissue integrity (157). Gene therapy restoring IDUA activity effectively clears systemic and skeletal GAG storage, improves joint stiffness, promotes near-normal bone growth, and stabilizes spinal architecture, demonstrating that recovering lysosomal function can re-establish the ECM-associated microenvironment and restore bone remodeling dynamics (157).

In summary, the lysosomal system exerts multidimensional control over ECM degradation, mineralization, and biomechanical performance through its enzymatic activity, acidification capacity, ion homeostasis, membrane trafficking, and metabolic load under pathological conditions. Its interplay with the ECM forms the core molecular network governing bone resorption. Even subtle perturbations in lysosomal function are sufficient to induce imbalanced resorption, aberrant matrix degradation, or mineralization defects, collectively driving the onset and progression of osteoporosis. Therefore, molecular targets along the lysosome–matrix interaction axis—such as CatK, RUFY4, and SNX10-associated trafficking machinery—represent promising strategies for precision intervention to normalize bone resorption and improve skeletal structural quality (**Table 2**).

**Table 2:** Interaction mechanisms between lysosomes and bone ECM in osteoporosis.

Bone cell type	Lysosome–ECM function in osteoporosis	Key mechanism (single item per row)	Reference
Osteoclast	ECM degradation	CatK inhibition blocks transition from pit-type to trench-type resorption.	(142)
Osteoclast	ECM degradation	Estrogen deficiency induces CTSK upregulation and collagen disorganization.	(143)
Osteoclast	ECM degradation	CTSK knockout increases BV/TV and trabecular thickness, showing impaired resorption.	(144)
Osteoclast	Clinical inhibition	Odanacatib reduces bone resorption markers and increases BMD.	(145, 146)
Osteoclast	CatK signaling	Odanacatib efficacy correlates with decreased CatK activity.	(147, 148)
Osteoclast	Pathological activation	CatK is persistently upregulated in osteoarthritic tissues.	(149)
Osteoclast	Pharmacologic inhibition	ONO-5334 shows sustained suppression of bone resorption.	(150, 151)
Osteoclast	Lysosomal trafficking	RUFY4 mediates lysosome transport to the ruffled border via RUN/FYVE domains.	(152)
Osteoclast		RANKL–NF-κB p65–NFATc1 induces RUFY4 expression.	(20)
Osteoclast	Membrane trafficking	SNX10 regulates vesicular trafficking and maintains ruffled border integrity.	(153)
Osteoclast	Acidification defects	SNX10/TCIRG1/CLCN7 mutations impair acidification and ruffled border formation.	(23, 79, 154, 155)
Osteoclast	Mineralization phenotype	TCIRG1 mutations cause osteoid accumulation and reduced mineralization.	(156)
Osteoblast	ECM–lysosome coupling	IDUA deficiency causes GAG accumulation and ECM disruption.	(157)
Osteoblast	ECM–lysosome signaling	Lysosomes integrate ECM biochemical and mechanical cues.	(158)
Osteoblast	Osteogenesis suppression	ECM disorganization reduces lysosomal acidification and trafficking.	(131, 159, 160)
Osteoblast	Energy signaling	DHAP lysosomal degradation releases PO_4_³^-^ → ATP → cAMP/PKA → Runx2.	(161)
Osteoblast	Senescence	CHMP5 loss disrupts endolysosomal trafficking and reduces VPS4A.	(162)
BMSC	Lysosomal acidification	TMEM175 deficiency reduces acidification and impairs osteogenic differentiation.	(15)
BMSC	Inorganic cue translation	Citrate–Zn–HA nanoparticles activate WNT pathway via lysosomal processing.	(163)
Osteocyte	Autophagy dependence	Punctate LC3 indicates basal autophagy requirement under stress.	(164)
Osteocyte	GC-induced autophagy	Glucocorticoids increase LC3A/B, ATG2, ATG7.	(165)
Osteocyte	Dose-dependent GC effects	Low-dose GC protective; high-dose blocks autophagosome–lysosome fusion.	(166)
Osteocyte	Matrix sensing	Autophagy impairment disrupts ECM sensing and mechanotransduction.	(65)
Osteocyte	PLR regulation	Lactation increases CTSK and activates PLR.	(70)
Osteocyte	Pathological integration	Lysosomal dysfunction causes defects in signaling and survival.	(168, 169)

CatK, Cathepsin K; CTSK, Cathepsin K Gene; ECM, Extracellular Matrix; BV/TV, Bone Volume/Tissue Volume; BMD, Bone Mineral Density; RANKL, Receptor Activator of Nuclear Factor κB Ligand; NF-κB, Nuclear Factor kappa-B; NFATc1, Nuclear Factor of Activated T Cells 1; RUFY4, RUN and FYVE Domain–Containing Protein 4; Rab7, Ras-Related Protein Rab-7; LAMP2, Lysosome-Associated Membrane Protein 2; SNX10, Sorting Nexin 10; TCIRG1, T Cell Immune Regulator 1 (V-ATPase Subunit); CLCN7, Chloride Channel 7; TNFRSF11A, Tumor Necrosis Factor Receptor Superfamily Member 11A; IDUA, α-L-Iduronidase; GAG, Glycosaminoglycan; MPSIH, Mucopolysaccharidosis Type I H; TMEM175, Transmembrane Protein 175; ATP, Adenosine Triphosphate; cAMP, Cyclic Adenosine Monophosphate; PO_4_³^-^, Phosphate Ion; CHMP5, Charged Multivesicular Body Protein 5; VPS4A, Vacuolar Protein Sorting–Associated Protein 4A; WNT, Wnt Signaling Pathway; HA, Hydroxyapatite; DHAP, Dihydroxyacetone Phosphate; PTH, Parathyroid Hormone; PTHrP, Parathyroid Hormone–Related Peptide; 1,25(OH)_2_D, 1,25-Dihydroxyvitamin D; ROS, Reactive Oxygen Species; GC, Glucocorticoid; LC3, Microtubule-Associated Protein 1A/1B-Light Chain 3; ATG2, Autophagy-Related Protein 2; ATG7, Autophagy-Related Protein 7; PLR, Perilacunar/Canalicular Remodeling; BMSC, Bone Marrow–Derived Mesenchymal Stem Cell; MVB, Multivesicular Body; MV, Mineralizing Vesicle.

### 4.2 Mechanisms underlying bone formation

The homeostasis of bone formation depends on the precise interplay between the ECM and the lysosomal system within osteogenic cells, an interaction that becomes markedly disrupted in osteoporosis. Recent studies position lysosomes as a central lysosomal signaling hub, integrating extracellular matrix cues with intracellular differentiation programs (158). Under osteoporotic conditions, alterations in ECM composition and mechanics, together with defects in lysosomal trafficking, acidification, and degradative efficiency, destabilize this regulatory axis and ultimately diminish osteogenic output (15).

As a biochemical and biomechanical information reservoir, the ECM modulates lysosomal behavior through integrin-dependent adhesion signaling, mechanotransduction, and matrix remodeling dynamics (170, 171). In osteoporosis, reduced matrix mineralization, disorganized collagen networks, and nanoscale structural deterioration impair osteoblast mechanosensitivity and disrupt lysosomal positioning, acidification, and autophagy–lysosome flux (131, 159, 160). For instance, TMEM175 deficiency in BMSCs leads to insufficient lysosomal acidification, compromising ECM signal responsiveness and resulting in pronounced defects in osteogenic differentiation (15). These findings demonstrate that pathological ECM remodeling can reprogram lysosomal biogenesis and function, thereby altering cellular metabolism and lineage commitment.

Lysosomes act as key executors of the osteogenic program by regulating autophagic flux, mitochondrial homeostasis, and organelle quality control (1). Beyond their canonical degradative role, lysosomes also function as metabolic and signaling transducers. Biomimetic citrate–zinc–HA nanoparticles internalized by BMSCs are selectively delivered into lysosomal compartments, where they enhance WNT activity, increase ALP expression, and promote ECM mineralization—highlighting lysosomal participation in translating inorganic microenvironmental cues into osteogenic signals (163). Similarly, DHAP undergoes lysosomal degradation to release PO_4_³^-^, driving local ATP production and activating the adenosine–cAMP/PKA–Runx2 cascade, further supporting the unique role of lysosomes in converting mineral inputs into bioenergetic and transcriptional outputs (161).

Structural disruption of the endolysosomal pathway has also emerged as a major driver of osteoblast senescence and functional exhaustion. Loss of CHMP5 perturbs endolysosomal trafficking and degradation, reduces VPS4A abundance, induces mitochondrial dysfunction, and promotes excessive ROS accumulation, collectively accelerating osteoblast senescence. Senescent cells not only lose osteogenic capacity but also impair the local bone microenvironment through paracrine signaling. Notably, senolytic clearance effectively reverses CHMP5-deficiency–associated defects in bone formation, underscoring the therapeutic potential of targeting the lysosome–senescence axis (162).

Together, ECM deterioration and lysosomal dysfunction establish a mutually reinforcing pathological loop. ECM mechanical weakening and compositional degradation suppress lysosomal activity, whereas lysosomal defects further disrupt ECM synthesis and mineralization, driving persistent declines in bone formation. Deeper mechanistic layers include the breakdown of ECM–integrin–lysosome positioning circuits, insufficient metabolic output from the autophagy–mitochondria axis, and ESCRT-dependent endolysosomal trafficking imbalances that promote cellular senescence. Disruption of this bidirectional ECM–lysosome communication constitutes a core pathogenic mechanism underlying osteoporotic bone loss (**Table 2**).

### 4.3 Dysregulation of osteocytes homeostasis

Osteocytes are the terminally differentiated cells of the osteoblastic lineage and represent the most abundant and long-lived cell population within bone tissue (9). Owing to their residence in a microenvironment characterized by hypoxia, nutrient limitation, and continuous mechanical loading, osteocytes are highly dependent on lysosome-mediated autophagy. This dependence not only ensures their survival and metabolic homeostasis but also directly shapes the quality and mechanical integrity of the bone matrix (168, 169). Osteocytes in mouse and human cortical bone display punctate LC3 localization, indicating that a basal level of autophagy is essential for sustaining osteocyte viability under nutrient deprivation or hypoxic stress (164). Beyond maintaining homeostasis under physiological conditions, osteocyte autophagy plays a crucial role in mediating endocrine and pharmacologic modulation of bone metabolism. Glucocorticoid (GC) exposure induces autophagosome accumulation and upregulates LC3A/LC3B, ATG2, ATG7, and other key components, reflecting robust activation of the lysosome-dependent autophagy pathway (165). This effect is dose-dependent—low-dose GC-induced autophagy enhances antioxidant defenses and confers partial protection to bone, whereas high doses suppress antioxidant gene expression, impair autophagosome–lysosome fusion and degradation, and ultimately trigger cellular stress, functional decline, and accelerated bone loss (166).

The dependence of osteocytes on lysosomal function is also evident in their intimate interaction with the bone matrix. Once lysosome–autophagy activity becomes impaired, osteocytes exhibit synchronized defects in matrix sensing, signal transmission, and survival, constituting an early pathogenic event in osteoporosis (1, 167). Under specific physiological stresses such as lactation, elevated calcium demand drives osteocytes to activate local perilacunar remodeling and upregulate bone-resorptive genes including CTSK (70). Genetic studies reveal that osteocyte-specific Ctsk deletion markedly suppresses lactation-induced expansion of perilacunar cavities, reduces osteoclast recruitment, and prevents deterioration of trabecular microarchitecture. Moreover, this manipulation stabilizes systemic calcium homeostasis by modulating PTH, PTHrP, and 1,25(OH)_2_D levels, thereby maintaining calcium content in both maternal serum and milk (70). These findings highlight that the osteocytic lysosomal system, beyond supporting intrinsic homeostasis, participates in local matrix degradation and systemic calcium regulation during complex physiological adaptations.

Collectively, the lysosome–autophagy axis that maintains osteocyte homeostasis occupies a central role in osteoporosis. As cells positioned at the intersection of skeletal structure, mechanoadaptation, and metabolic regulation, osteocytes translate lysosomal dysfunction into multilevel disturbances that cumulatively drive bone deterioration. Conceptualizing the lysosome–matrix interface as a key pathogenic node not only deepens our understanding of the systemic nature of osteoporosis but also provides a new therapeutic framework for targeting osteocyte autophagy and lysosomal function in future interventions (**Table 2**).

## 5 Intervention strategy targeting the ECM-lysosomal axis

### 5.1 Restoring and enhancing lysosomal function: organelle-targeted therapeutic interventions

Restoring lysosomal function is fundamental for sustaining ECM turnover and skeletal homeostasis (16), positioning reinforcement of the “ECM–lysosome axis” as a central therapeutic direction. As the master regulator of lysosomal biogenesis and the autophagy–lysosome system, TFEB has emerged as one of the most compelling organelle-level targets with translational potential (44). In a CRISPR-activation model (Tfeb^CRa), elevation of endogenous TFEB in osteoblasts increased lysosomal abundance, augmented autophagic flux, and enhanced matrix remodeling capacity, ultimately leading to substantial gains in bone mass and mechanical strength (19). These findings establish a strong mechanistic rationale for employing pharmacological agents, small molecules, or gene modulators to activate TFEB as a direct route to reconstitute osteoblastic lysosomal competence and promote bone anabolism.

Within osteoclasts, the RANKL–PKCβ–TFEB signaling axis similarly governs lysosomal biogenesis and offers an attractive window for intervention (94). Modulating this cascade to curb pathological lysosomal expansion may effectively restrain excessive bone resorption while preserving osteoclast metabolic stability (94). Lysosomal dysfunction often accelerates skeletal aging, as SASP derived from senescent osteoblasts and stromal cells degrades ECM integrity and suppresses lysosomal renewal (100). Targeting these senescent populations—either through senolytics to eliminate them or senomorphics to mitigate SASP—has the potential to re-establish ECM quality, relieve inflammatory repression of lysosomal biogenesis, and restore the positive regulatory loop between ECM and lysosomes (172). Beyond intrinsic skeletal cells, tuning the secretory phenotypes of immune cells such as macrophages and T cells toward reparative states may further enhance lysosomal fitness, thereby broadening therapeutic leverage across the ECM–lysosome axis (173, 174).

Regulation of lysosomal trafficking and maturation in osteoclasts also represents a therapeutically powerful strategy. RUFY4, a Rab7–LAMP2 adaptor coordinating late endosome–lysosome fusion, is indispensable for lysosomal maturation (20). Loss of Rufy4 disrupts CatK delivery to the bone interface and diminishes bone resorption without compromising osteoclast viability or differentiation (20). Evidence from OVX and inflammatory high–bone turnover models demonstrates that Rufy4 deficiency preserves trabecular architecture *in vivo* (175). This mechanism of “selectively blocking lysosomal secretion” offers a refined approach to suppress bone resorption while avoiding pathological lysosomal accumulation. CatK, the predominant lysosome-derived collagenase responsible for type I collagen degradation, remains a major focus of therapeutic development (176). Although classical CatK inhibitors reduce resorption, adverse effects and off-target activities have limited their clinical success (176). Consequently, a new paradigm emphasizing “function-selective inhibition” has emerged, aiming to suppress CatK’s collagenolytic activity while retaining its intracellular proteolytic functions (177). Approaches such as heparan sulfate–mediated CatK dimerization, allosteric inhibitors, and tanshinone-derived natural compounds have demonstrated efficacy in reducing bone resorption while minimizing lysosomal stress (177, 178). Additionally, the non-pyroptotic action of GSDMD—through its p20 fragment binding PI (3)P and thereby hindering endosomal maturation and CatK secretion—illustrates that modulating lysosomal maturation cascades can serve as an indirect yet potent means to regulate CatK activity (179).

Taken together, strategies that boost lysosomal biogenesis in osteoblasts, constrain lysosomal secretion in osteoclasts, eliminate lysosome-defective senescent cells, and achieve function-selective modulation of CatK all converge toward a common goal: rebalancing the ECM–lysosome axis and restoring skeletal homeostasis at the organelle level. These organelle-centric interventions not only reinforce the regenerative capacity of the ECM but also establish a mechanistic framework for designing next-generation osteoporosis therapies that are both more precise and safer (**Table 3**).

**Table 3:** Intervention strategy targeting the ECM–lysosomal axis.

Intervention category	Cell/target	Therapeutic direction	Key mechanism (single item per row)	Reference
Lysosomal Function	Osteoblast	Enhance lysosomal biogenesis	TFEB activation increases lysosomal abundance and autophagic flux.	(44, 47)
Lysosomal Function	Osteoclast	Control lysosomal expansion	RANKL–PKCβ–TFEB axis regulates lysosomal biogenesis in osteoclasts.	(94)
Lysosomal Function	Senescent cells	Restore ECM quality	Clearing senescent osteoblasts/stromal cells via senolytics removes SASP burden.	(100, 172)
Lysosomal Function	Immune cells	Promote reparative immunity	Modulating macrophage/T-cell secretory phenotype enhances lysosomal fitness.	(173, 174)
Lysosomal Function	Osteoclast	Reduce CatK secretion	RUFY4 deficiency reduces CatK trafficking to bone surface.	(20, 175)
Lysosomal Function	CatK enzyme	Reduce collagenolysis	Function-selective inhibition suppresses CatK collagenase activity while preserving intracellular proteolysis.	(177)
Lysosomal Function	CatK enzyme	Natural inhibitors	Tanshinone-derived compounds suppress CatK with lower lysosomal toxicity.	(178)
Lysosomal Function	Osteoclast	Indirect CatK suppression	GSDMD-p20 binds PI (3)P and inhibits endosomal maturation → reduces CatK secretion.	(179)
ECM Repair	ECM	Restore ECM structural integrity	ECM damage initiates osteoporosis pathology.	(180)
ECM Repair	Bone cells	Improve matrix mechanosensing	ECM deterioration weakens lysosome-dependent mechanosensing & cargo recycling.	(131)
ECM Repair	ECM (collagen)	Promote collagen crosslinking	LOX/LOXL2 catalyze pyridinoline crosslink formation, strengthening the ECM.	(181)
ECM Repair	ECM–lysosome axis	Restore lysosomal biogenesis	OLR1/LOX-1 activation retains TFEB in cytosol → suppresses lysosomal formation.	(182)
ECM Repair	ECM mechanics	Improve mechanosensitivity	WBV enhances lysosomal activity and autophagy across bone cells.	(183)
ECM Repair	Clinical (bone tissue)	Increase BMD	High-frequency low-intensity WBV increases BMD in postmenopausal women.	(184)
Nanomaterials	BMSC	Activate osteogenesis	HAp/ion-based nanocarriers accumulate in lysosomes and activate osteogenic pathways.	(185)
Nanomaterials	BMSC	Ion-triggered lysosomal activation	cit-Zn-HAp releases Zn²^+^/Ca²^+^ in lysosomes → activates WNT/β-catenin/Runx2.	(163)
Nanomaterials	Bone cells	pH-responsive drug release	Acid-responsive nanocarriers restore lysosomal acidification & autophagy.	(186)
Nanomaterials	Bone tissue	Improve targeting & safety	Lysosome-targeted nanodelivery enhances efficacy & reduces systemic toxicity.	(185, 186)

TFEB, Transcription Factor EB; PKCβ, Protein Kinase C beta; SASP, Senescence-Associated Secretory Phenotype; RANKL, Receptor Activator of Nuclear Factor-κB Ligand; NF-κB, Nuclear Factor kappa-light-chain-enhancer of activated B cells; Rab7, Ras-related protein Rab-7; LAMP2, Lysosome-Associated Membrane Protein 2; CatK/CTSK, Cathepsin K; OVX, Ovariectomy (model); GSDMD, Gasdermin D; PI(3)P, Phosphatidylinositol-3-phosphate; ECM, Extracellular Matrix; LOX, Lysyl Oxidase; LOXL2, Lysyl Oxidase-Like Protein 2; OLR1/LOX-1, Oxidized Low-Density Lipoprotein Receptor 1; LIPUS, Low-Intensity Pulsed Ultrasound; WBV, Whole-Body Vibration; BMD, Bone Mineral Density; BMSC, Bone Marrow Mesenchymal Stem Cell; HAp, Hydroxyapatite; WNT, Wingless/Integrated Signaling Pathway; ALP, Alkaline Phosphatase; ZIF, Zeolitic Imidazolate Framework; CZA, Curcumin-Loaded ZIF Nanoparticles; NP, Nanoparticle.

### 5.2 Reconstructing the extracellular matrix: matrix-directed repair strategies

Damage to the ECM is frequently an initiating event in osteoporosis (180), and disruptions in ECM composition and structural organization further weaken the lysosome-dependent matrix sensing, cargo recycling, and remodeling capacities of osteoblasts, osteoclasts, and osteocytes (131). Within this conceptual framework, ECM deterioration not only provides the material basis for loss of mechanical strength but also marks the initial point of imbalance within the “ECM–lysosome axis.” Consequently, restoring ECM architecture and function represents a crucial therapeutic opportunity for reversing bone loss and reactivating intracellular–extracellular homeostatic circuits.

Among available strategies, targeting collagen crosslinking is recognized as a central approach to reinforcing the mechanical continuity of the bone matrix (181). The LOX/LOXL2 family, which catalyzes the formation of mature pyridinoline crosslinks, not only governs ECM stability but also modulates the lysosomal metabolic state of osteoblasts. Exogenous supplementation of LOX/LOXL2 has been shown to markedly enhance ECM maturation and structural organization in engineered bone constructs, whereas LOX inhibition disrupts osteogenic differentiation and reduces mineralization efficiency (181). These findings underscore the essential role of promoting physiological crosslink formation to preserve ECM integrity. Importantly, ECM repair is tightly coupled to lysosomal regulation at the molecular level. For instance, activation of OLR1/LOX-1 retains TFEB in the cytosol, thereby suppressing autophagy–lysosome biogenesis (182), highlighting a bidirectional causal relationship between impaired ECM sensing and lysosomal dysfunction.

Beyond biochemical remodeling, strategies aimed at reinforcing the physical attributes of the ECM have also gained traction. Mechanical stimulation via low-intensity pulsed ultrasound (LIPUS) or whole-body vibration (WBV) increases local matrix stiffness, enhances lysosome formation, and augments autophagic flux (182, 183). Such interventions improve mechanosensitivity across bone cells in experimental settings and have shown promising clinical benefits. Notably, high-frequency, low-intensity WBV administered at adequate cumulative doses significantly increases bone mineral density in postmenopausal women (184), providing early evidence for mechanical enhancement as a viable therapeutic avenue.

Collectively, approaches ranging from promoting collagen crosslinking to strengthening ECM mechanical properties converge on the same therapeutic objective: rebuilding the “ECM–lysosome positive feedback loop” that underlies skeletal homeostasis. By restoring ECM structural integrity and reinforcing lysosome-dependent metabolic and mechanosensitive pathways, these matrix-oriented strategies offer a mechanistically grounded and translationally promising direction for combating osteoporosis (**Table 3**).

### 5.3 Nanomaterial-based therapies and drug delivery systems

Nanomaterials provide a highly tunable platform for precise manipulation of the ECM–lysosome axis, enabling simultaneous remodeling of the bone microenvironment and intracellular pathways through organelle-targeted delivery. Recent advances demonstrate that nanocarriers based on hydroxyapatite (HAp), metal ions, or biomimetic ECM structures are actively internalized by BMSCs and other bone-related cells, accumulating within endosome–lysosome compartments where they initiate osteogenic signaling cascades (185). For example, cit-Zn-HAp nanoparticles gradually release Zn²^+^ and Ca²^+^ in the acidic lysosomal milieu, stimulating WNT/β-catenin and Runx2 signaling, increasing ALP activity, and enhancing mineralization. These findings indicate that “lysosome-localized ionic modulation” effectively restores disrupted ECM–lysosome coupling (163).Similarly, acid-responsive or pH-sensitive nanocarriers exploit the low pH of lysosomes to achieve precise release of drugs, transcriptional regulators, or autophagy–lysosome modulators, thereby improving lysosomal acidification, augmenting autophagic flux, or restoring ECM turnover (186). Acid-responsive ZIF nanoparticles, for instance, successfully deliver curcumin to bone tissue, and intravenous administration of CZA reduces bone loss in OVX mice (186). By targeting intracellular organelles, these systems not only improve therapeutic efficacy but also minimize nonspecific toxicity associated with systemic drug exposure—making them one of the most promising strategies for modulating the ECM–lysosome axis. Expanding beyond small molecules, both viral and non-viral nanocarriers have been employed for bone-targeted gene delivery to correct congenital lysosome–mineralization defects or potentiate osteogenic pathways. Nevertheless, limited vascularity and the dense ECM structure of bone continue to restrict intratissue drug penetration. Future efforts should focus on optimizing particle size, surface charge, ECM-binding affinity, and lysosomal activation mechanisms to maximize delivery efficiency and achieve sustained regeneration within the bone-defect microenvironment (**Table 3**).

## 6 Limitations and prospects

This review centers on the core pathological basis of osteoporosis, beginning with the pressing reality of its global epidemiology and advancing a conceptual framework that positions “ECM–lysosome crosstalk” as a fundamental determinant of skeletal homeostasis. The structural integrity and dynamic renewal of the bone ECM are essential for maintaining mechanical competence and cellular functionality, while lysosomes—through acidification, proteolysis, autophagy flux regulation, and inter-organelle metabolic coupling—govern ECM turnover and microenvironmental equilibrium. Here, we systematically delineate the lysosome-dependent roles of osteoclasts, osteoblasts, and osteocytes in ECM regulation, emphasizing how the biochemical composition, mechanical properties, and nanoscale topology of the ECM profoundly shape lysosomal biogenesis, functional states, and signaling outputs. Disruption of this bidirectional ECM–lysosome axis is pervasive across the onset and progression of osteoporosis, manifested by heightened osteoclast acidification and secretion, impaired autophagy–mitochondrial coupling in osteoblasts, and collapse of osteocytic PLR and the lacunar–canalicular system—all highlighting the centrality of this signaling network.

Despite growing interest in ECM–lysosome crosstalk, major mechanistic gaps remain. The temporal and spatial specificity with which lysosomal dynamics participate in lineage differentiation and matrix remodeling is still insufficiently defined. Most current evidence derives from cell-based or animal studies, and translation into clinically actionable biomarkers or drug targets remains limited. Moreover, the principles underlying intercellular lysosomal communication—such as cross-talk between osteoblasts and osteoclasts or between BMSCs and immune cells—are only beginning to be uncovered. Future research would benefit from integrating spatial transcriptomics, single-cell multi-omics, and high-resolution imaging to construct a more comprehensive spatiotemporal regulatory map. On the therapeutic front, agents targeting lysosomal function, including small molecules and nanodelivery platforms such as pH-responsive carriers and lysosome-stabilizing compounds, are emerging as promising interventions. Recent studies demonstrate that modulating TFEB nuclear translocation, inhibiting mTOR, or deploying acid-stabilizing agents can markedly attenuate osteoclast hyperactivation and rescue impaired osteogenesis, underscoring strong translational potential.

Taken together, ECM–lysosome crosstalk represents a pivotal bridge linking cellular metabolism with matrix remodeling, and its conceptual and mechanistic maturation holds substantial promise for advancing osteoporosis research and treatment. Continued interdisciplinary collaboration—integrating materials science, systems biology, and nanotechnology—will be essential for elucidating the molecular foundations, tissue-specific properties, and clinical relevance of this crosstalk. Such efforts are expected to provide a robust foundation for precision prevention and individualized therapy in osteoporosis.
